# Comparison of PuraStat self-assembling peptide hydrogel versus mineral-based Hemospray for endoscopic hemostasis of upper and lower gastrointestinal lesions in pigs

**DOI:** 10.3389/fgstr.2022.971353

**Published:** 2022-08-12

**Authors:** Eun Seok Gil, Kate O’Neill, Elton Aleksi, Jay Budrewicz, Raffaele Melidone, Lisa Spirio

**Affiliations:** ^1^ 3-D Matrix Inc., Newton, MA, United States; ^2^ 3-D Matrix Ltd., London, United Kingdom; ^3^ CBSET Inc., Department of Pathology, Lexington, MA, United States; ^4^ First Edge Consulting LLC, Weymouth, MA, United States

**Keywords:** nanofiber, RADA16, PuraStat, Hemospray, gastrointestinal hemostasis, wound healing, endoscopic surgery, self-assembling peptide

## Abstract

**Objective:**

To compare a RADA16-based self-assembling peptide hydrogel versus an inorganic powder-based spray device for controlling postoperative bleeding in upper and lower GI mucosal lesions in pigs.

**Methods:**

Multiple mucosal lesions were endoscopically-created in the stomachs and lower colons of six Yorkshire swine on Day 0. Three animals’ wounds were treated with 2.5% RADA16 solution (PuraStat^®^), two animals were treated with an aerosolized mineral powder (Hemospray^®^), and one animal was an untreated control. Primary outcomes were test article applications required to control initial bleeding, time-to-hemostasis, and rebleeding incidence. Secondary outcomes included animal recovery, and clinical pathology at weekly endoscopic evaluations and the 4-week study terminus.

**Results:**

Number of material administrations required and time-to-hemostasis was comparable between PuraStat and Hemospray groups. Rebleeding rates were comparable between treatments. Two of 12 (17%) Hemospray and none of 18 (0%) PuraStat stomach sites experienced rebleeding during the final 4 min of the 10-min observation period. No delayed bleeding was observed during weekly endoscopic follow-ups. Hematology and serology values remained normal in all animals. Histology showed expected healing responses at all PuraStat- and Hemospray-treated defects, with less inflammation than untreated sites. Histomorphological observations were comparable between different groups for both the stomach and colon for test and control materials, with lower inflammation scores than untreated sites. Performance and usability responses were generally good with both systems, although the Ability to Treat Intended Site score was significantly better with PuraStat in upper GI lesions.

**Conclusions:**

PuraStat and Hemospray were effective topical hemostats for mild-to-moderate bleeding in upper and lower GI wounds. Rebleeding was observed in two of 12 Hemospray-treated sites and none of 18 PuraStat-treated sites. PuraStat and Hemospray were associated with better wound healing than untreated controls. The ability to treat upper GI lesions was easier with the PuraStat versus Hemospray system.

## Introduction

Bleeding in the gastrointestinal (GI) tract is a leading cause of morbidity and is associated with an estimated 20,000 deaths per year in the United States alone (1). In any surgical procedure, hemostasis is vital to success and in some cases bleeding can be life threatening. Therefore, fast and effective bleeding management is essential for achieving optimal patient outcomes. Most current GI hemostasis therapies rely on thermocoagulation (*e.g.*, electrocautery), mechanical (*e.g.*, hemoclip), and injection (*e.g.*, epinephrine) devices (2). These therapies carry risk of damage to the surrounding tissue, including perforation, and require technical skill to precisely place the device on the bleeding site and avoid complications.

**Figure 1:**
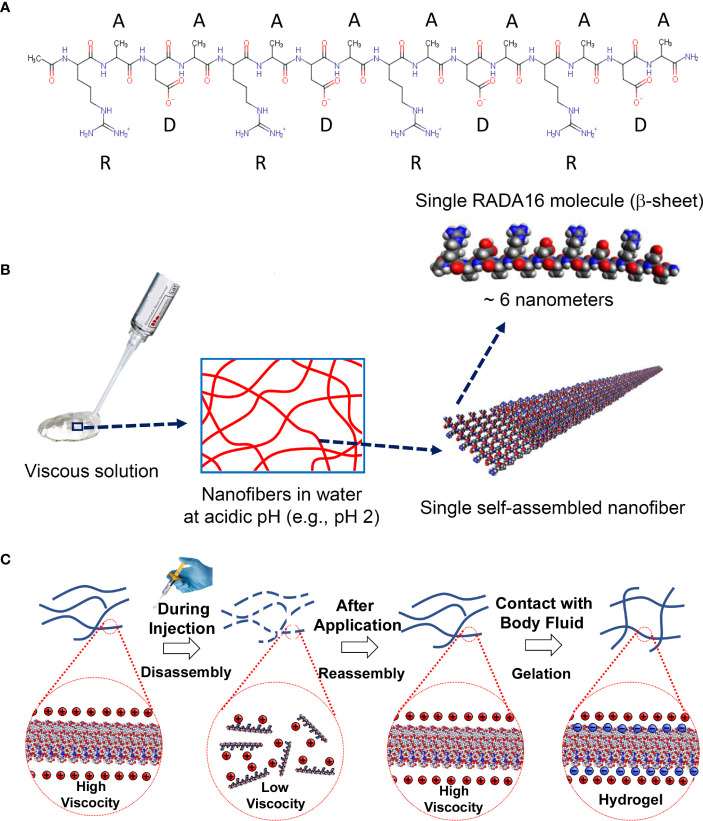
Chemical structure and self-assembly of RADA16 into higher-order hydrogels. **(A)** The RADA16 peptide of PuraStat has 16 amino acids organized as repeated 4-amino acid sequences containing “R” (positively-charged arginine), “A” (hydrophobic alanine), and “D” (negatively-charged aspartic acid) residues. **(B)** RADA16 undergoes spontaneous and revisable self-assembly in acidic solutions to generate nanofibers. RADA16 molecules with β-sheet conformation interact through face-to-face hydrophobic interactions and edge-to-edge hydrogen bonding to form layered and extended nanofibers, ≈6 nm wide. These extracellular matrix-like nanofibers form a viscous and transparent aqueous solution at a relatively low concentration range (*e.g.*, 0.1~2.5% weight/volume). **(C)** Illustration of RADA16 structure and properties as it is applied to and gels on a wound site. Acidic aqueous solutions of RADA16 are viscous and exhibit thixotropic disassembly/reassembly, which allows their easy administration to wound sites through catheters and syringes, with viscosity returning immediately after administration. Upon contact with the physiological pH of body fluids including blood, the net surface charge of RADA16 nanofibers become zero resulting in the physical crosslinking by hydrophobic interactions between neighboring RADA16 nanofibers, so that RADA16 solution forms *in-situ* hydrogels on the wound site and act as a physical adhesive that is hemostatic and supports wound healing. Adopted from Sankar *et al.*, *Front. Bioeng. Biotechnol*. 2021; doi: 10.3389/fbioe.2021.679525, in accordance with Creative Commons Attribution License CC-BY.

Topical hemostatic agents provide a minimally-invasive approach to endoscopically achieving hemostasis at bleeding sites within the GI tract (2). Current approaches include trans-endoscope delivery of coagulant mineral-based or organic powders as aerosols, and hemostatic liquid formulations that polymerize to form a barrier upon contact with bleeding GI tissues (2–5). One powder formulation, Hemospray^®^ (Cook Medical, Bloomington, IN) is an inorganic, nonabsorbable, biologically inert mineral (bentonite) powder that is aerosolized using compressed CO_2_ for catheter delivery through the endoscope instrument channel (6). Hemospray absorbs water from blood and swells to form a temporary barrier that seals injured blood vessels; it sloughs off for fecal elimination within a few days. PuraStat^®^ is a viscous aqueous formulation containing monomers of the synthetic self-assembling peptide RADA16 that is also delivered to wound sites by a catheter through the endoscope (5). RADA16 is a linear oligopeptide containing 16 amino acids as repeated 4-amino acid sequences containing R (positively-charged arginine), A (hydrophobic alanine), and D (negatively-charged aspartic acid) residues (5, 7, 8). Upon contact with physiological pH and ion-rich blood, RADA16 rapidly self-polymerizes into a transparent hydrogel that acts as a barrier to bleeding (**Figure 1**). The polymerized gel is gradually hydrolyzed to non-toxic metabolites and disappears from the application site. Accumulating evidence suggest that in addition to staunching bleeding, the RADA16 hydrogel may also function as a permissive environment that promotes wound healing (5).

PuraStat and Hemospray both have demonstrated safety and utility as effective hemostats in animal studies and clinical trials, and both are approved for controlling GI bleeding in humans (4–11). However, to our knowledge no direct comparisons have ever been performed between these two formulations. The current study used a porcine model to directly compare PuraStat and Hemospray effects on primary hemostasis, rebleeding rates, and wound healing in experimentally created lesions in the upper and lower GI tracts.

## Materials and methods

### Study design and ethical oversight

This was a prospective, randomized, assessor-blinded, controlled study developed in compliance with International Standard ISO 10993-1 “*Biological evaluation of medical devices*” (12), and conducted in accordance with World Health Organization’s “*Good Laboratory Practices Handbook*” (13). Conditions were overseen by the CBSET Inc., Contract Research Organization’s (Lexington, MA) Institutional Animal Care and Use Committee (IACUC), and conformed to the “*Guide for the Care and Use of Laboratory Animals, 8th edition*” (14), and the ARRIVE 2.0 Guidelines (15). CBSET is an AAALAC International accredited facility and is registered with the U.S. Department of Agriculture.

### Test materials

Hemospray^®^ (Cook Medical, Bloomington, IN) is an inorganic, nonabsorbable, mineral powder (6), and PuraStat-GI^®^ (3-D Matrix Inc., Newton, MA) is a 2.5% aqueous solution of RADA16 (7, 8). Both formulations are commercially available as sterile ready-to-use preparations packaged with their own trans-endoscopic catheter delivery systems.

### Animal care and preparation

The healthy swine model was chosen as the experimental species for this study because the size and anatomy of the gastrointestinal system is clinically relevant for testing human gastrointestinal medical treatments, and its established use for evaluating topical GI hemostats (5, 16–23). Six adolescent male Yorkshire swine (41.7−51.9 kg) were purchased from Animal Biotech Industries (Danboro, PA) and acclimatized for ≥12 days before undergoing procedures. Swine were offered Purina Lab Diet (#5084 Laboratory Porcine Diet Grower) once daily, until start of a liquid diet 3 days prior to surgery, through study terminus. Liquid diet contained: #5084 Laboratory Porcine Diet Grower, full-fat yogurt, high-calorie supplemental drink (*e.g.*, Boost), whey protein and amino acid powder, and was provided at least once per day, divided into multiple feedings if needed. Water was provided *ad libitum* throughout the study. Animals were fasted for 24h prior to surgery, with colon cleansing performed by addition of magnesium citrate and Miralax to the last pre-operative meal, and enema administration.

### Anesthesia

Animals were allocated to PuraStat, Hemospray, or sham surgery/no-treatment Control groups by random number generation. Tiletamine-zolazepam (50:50 mass ratio; 4–6 mg/kg, IM) was administered as a pre-anesthetic. Isoflurane (in 100% oxygen) was administered to effect *via* mask/nosecone until animals were sufficiently anesthetized to allow endotracheal intubation and surgery. Peripheral vein access allowed supportive IV fluid delivery. Ophthalmic lubricant was applied to the eyes. Pre-emptive analgesia, buprenorphine (0.01 mg/kg, IM), was administered at anesthesia/surgical induction (prior to surgical incision). Positive-pressure mechanical ventilation was maintained for the procedure duration. Electrocardiogram, pulse, respiratory rate, SpO_2_, and temperature were monitored during surgery. Warm water heating pads and/or other warming devices were used to maintain body temperature while anesthetized.

### Surgical procedures

Animals were placed in dorsal recumbency or lateral recumbency per endoscopist discretion, and the GI tract (stomach and colon) was accessed using an endoscope (**Table 1**). CO_2_ insufflation was used distend the lumen to facilitate visualization. A single endoscopist (HA) performed all study surgeries and was not blinded to treatment due to the nature of the test articles. A histopathological analysis of wound tissues was performed in an assessor-blinded fashion.

**Table 1:** Animal allocation and mucosectomy/ESD lesion sites.

Animal	Pyloric Antrum	Gastric Body	Fundus	Distal Colon	Mid Colon	Proximal Colon
53647	Mucosectomy Ulcer	Mucosectomy Ulcer	Mucosectomy Ulcer	Mucosectomy Ulcer	Mucosectomy Ulcer	Mucosectomy Ulcer
53648	Mucosectomy Ulcer	Mucosectomy Ulcer	Mucosectomy Ulcer	Mucosectomy Ulcer	Mucosectomy Ulcer	Mucosectomy Ulcer
53651	ESD Ulcer	Mucosectomy Ulcer	Mucosectomy Ulcer	ESD Ulcer	Mucosectomy Ulcer	Mucosectomy Ulcer
53652	ESD Ulcer	Mucosectomy Ulcer	Mucosectomy Ulcer	ESD Ulcer	Mucosectomy Ulcer	Mucosectomy Ulcer
53653	ESD Ulcer	Mucosectomy Ulcer	Mucosectomy Ulcer	ESD Ulcer	Mucosectomy Ulcer	Mucosectomy Ulcer
53650	ESD Ulcer	N/A	ESD Ulcer	ESD Ulcer	N/A	ESD Ulcer
Treatment Color Key						
Group 1 = Hemospray^®^						
Group 2 = PuraStat^®^						
Group 3 = Untreated						

“ESD”, Endoscopic submucosal dissection.

#### Mucosectomy

A dual-channel gastroscope was used to access the stomach or colon. In the colon, a 26-gauge needle was used to inject a suitable volume of sub-mucosal injection solution (*i.e.*, BSC ORISE™ gel; Boston Scientific Corp., Marlborough, MA) into the submucosa at the target site to obtain sufficient submucosal lift. A grasper was passed down one endoscope working channel and a cold snare down the second channel. Target tissue was grasped and snared to perform multiple mucosectomies at the same site and create multifocal active bleeding gastric or colonic lesions. Four-to-six active gastric or colonic bleeds were created per animal. Defects were tattooed for identification at relooks and termination. Bleeding severity was graded per **Table 2** criteria. Bleeding rate was categorized by the endoscopist on a 0−4 point grading scale, with 0=None, 1=Mild, 2=Moderate, 3=Marked, and 4=Severe/Spurting. Each animal received either PuraStat or active comparator Hemospray administration over lesion sites (**Table 1**); each animal received only one treatment type. Bleeding duration was timed from active bleed start and PuraStat or Hemospray administration until hemostasis, declared as Time-to-Hemostasis, TTH. Wounds were assessed for 10 min, with the first evaluation within 3 min. If bleeding had not stopped or began again by 3 min, then additional material was applied and bleeding re-assessed after another 3 min. If the 2^nd^ application did not achieve hemostasis, then a 3^rd^ (final) application was made and the TTH assessed during the next 4 min. Rebleeding was assessed endoscopically in each animal after the last resection procedure in upper or lower GI, but prior to recovery. Bleeding observed at the 10-min observation point or after 3 applications of test article during this window was considered a treatment failure. Endoscopic images of defects were captured on video.

**Table 2:** Bleed Grading Criteria.

Grade	Bleeding Observation
**0**	None. No signs of active blood flow
**1**	Mild. Cherry red low oozing flow rate
**2**	Moderate. Moderate oozing flow rate
**3**	Marked. Fast oozing flow rate
**4**	Severe. Spurting flow

#### ESD

Electrocautery marked a ≈2×2 cm area in either the stomach or colon that served as margins of the simulated lesion. Defects were tattooed for identification, and a submucosal cushion was generated at the target site by BSC ORISE™ gel injection. *En bloc* mucosal resection of the simulated lesion to the level of the submucosa was performed using an endoscopic knife. After tissue retrieval, for test animals, the site was abraded using a rat-toothed grasper to create bleeding. Control animals did not have a bleed created. Endoscopic images of defects were captured on video. Duration of active bleeding was timed and blood flow rate was noted with the same criteria used for mucosectomy procedures (**Table 2**). Test animal ESD sites received PuraStat treatment and Control sites were untreated. In the event of severe spurting bleeding (grade 4), electrocautery (hemostatic forceps) was authorized for the bleeding point, and that lesion was excluded from analysis. Each wound site was observed for ≥10 min to assess TTH and rebleeding ≈3 min after wound creation/PuraStat application. If rebleeding was observed at either the 3- or 6-min checkpoint after first PuraStat application, an additional PuraStat application was performed and hemostasis or bleeding tracked. If bleeding was noted after a maximum of three permitted PuraStat applications or at the 10-min observation terminus, the intervention was deemed a failure. Notes were taken on the endoscopist’s assessment of device usability and performance.

### Postoperative care

After recovery from anesthesia, food and water were offered to the sheep. Buprenorphine (0.01 mg/kg, IM) was administered every 12h for analgesia through ≥24 postoperative hours. Omeprazole (20 mg, PO) was administered once daily from Days 1−14. Health checks were performed twice daily and clinical observations logged pre-operatively, once daily for the first 14 postoperative days, once per week thereafter, and on the day of necropsy. Body weights were recorded and blood was analyzed for hematology, clinical chemistry, and coagulation parameters pre-operatively and weekly after index surgery.

### Endoscopic relooks

Endoscopic reassessment of resection sites occurred on Days 7 ± 1, 14 ± 1, and 21 ± 1. Animals were fasted, colon-cleansed, and anesthetized per index surgery technique, and at least two images of each lesion site were endoscopically captured. Animals were recovered without supplemental buprenorphine administration.

### Terminal procedures

Animals were tranquilized with tiletamine-zolazepam (4–6 mg/kg, IM), anesthetized using isoflurane inhalant, and euthanized by peripheral venous overdose of KCl solution, per American Veterinary Medical Association guidelines (24). Comprehensive necropsy was conducted on all animals. Stomachs and descending colons were explanted and incised longitudinally to expose the lumen. Macroscopic images (mucosal and serosal) were taken of each treatment site. Excised organs were stretched on a flat plastic or cork plate prior and fixed in 10% neutral-buffered formalin. Full-thickness samples were excised from lesion centers, immersion-fixed in formalin, and processed for light microscopic characterization of inflammation and epithelialization, each graded on 0−4 scales (**Table 3**).

**Table 3:** Histopathology Grading Parameters.

Score	Inflammation
0	None.
1	Minimal, rare inflammatory cells, ≤5 per high-power field.
2	Mild, scattered inflammatory cells, ≤15 per high-power field.
3	Moderate, multifocal, notable infiltrate.
4	Extensive, packed sheets.
**Score**	**Epithelialization**
0	None; absence of mucosal epithelium.
1	Minimal, surface epithelium involving peripheral ≤10% of the defect.
2	Mild, mucosal re-epithelialization involving ≤50% of the defect.
3	Moderate, surface epithelium involving up to 90% of the defect.
4	Complete and mature epithelialization.

### System performance/usability

Hemospray and PuraStat delivery systems were user-evaluated for acute performance on a Pass/Fail basis. Performance characteristics included: satisfactory delivery through endoscope working channel, product did not clog/block application catheter, ease of visualization, and device malfunction.

Each Test/Control Article was evaluated for usability characteristics on a 0−4 scoring basis, whereby: 0 = Physician User has no difficulty completing procedural step; 1 = minimal difficulty resulting in longer procedure or additional instruction required, 2 = moderate difficulty resulting in either a much longer procedure or significantly more instruction required, 3 = inability to complete the procedural step or damage to the application system.

### Statistical analysis

Data are presented as mean ± SD or n (% of group), as indicated. Continuous variables were evaluated for normality and variance equivalence. When these conditions were met, a t-test was conducted; otherwise a Mann-Whitney Rank Sum Test was performed. Categorical data were evaluated using Fisher exact test. Two-tailed p-values <0.05 were considered statistically significant. Prism v.5.03 statistical and graphing software (GraphPad Software Inc., San Diego, CA) and SigmaPlot v.11 (Systat Software Inc., San Jose, CA) were used.

## Results

### Experimental overview and surgical approach

Mucosectomy defect healing compared effects of PuraStat versus Hemospray. Endoscopic submucosal dissection (ESD) defect healing compared effects of PuraStat versus untreated Control sites. Each animal received only one treatment. Six pigs were randomized to PuraStat (n=3), Hemospray active control (n=2), or Control (sham operated/no topical hemostat; n=1) groups. In each of the PuraStat animals, two 10-mm mucosectomy ulcers and one larger 20×20-mm ESD lesion were created each in the stomach and colon (*i.e.*, 6 wounds/animal). This provided a total of 12 mucosectomy and 6 ESD ulcers in the PuraStat group, evenly divided between stomach and colon. In the Hemospray group, 3 mucosectomy ulcers were created in both the stomach and in the lower colon, totaling 6 mucosectomy wounds per animal, for a total of 12 ulcers for direct comparison to the 12 mucosectomy sites in the PuraStat group. No ESD lesions were created in the Hemospray group. The single untreated Control animal received 2 ESD procedures each in the stomach and colon, for comparison to ESD lesions in PuraStat-treated pigs. Animal allocation and specific wound sites are provided in **Table 1**.

### General observations

No animal died or experienced a serious adverse event (AE) before scheduled euthanasia at postoperative 28 days. Adolescent animals gained a median 45% (range 36−53%) of their starting body weight through the 28-day study period. PuraStat-treated animals increased from a mean 48.3 ± 3.6 kg at Day 0 to 67.5 ± 5.9 kg at Day 28; Hemospray-treated animals increased from 46.9 ± 5.2 kg to 70.1 ± 9.6 kg during this time. There were no remarkable changes in hematology, serum chemistry, or fibrinogen values for animals during the study. Values for individual animals were generally within normal limits.

### Mucosectomy outcomes

#### Lesion size & bleeding severity

All 24 mucosectomy sites created in all animals were estimated to be uniformly ≈10 mm in greatest cross-sectional diameter. Initial bleeding scores in mucosectomy wounds were primarily Grade-1 (mild flow; 5/12 lesions in stomachs and 9/12 in colons) and Grade-2 (moderate flow; 6/12 lesions in stomachs and 3/12 in colons); only a single instance of Grade-3 bleeding was observed, in a stomach lesion. Although we observed more ≥Grade-2 bleeding in upper GI wounds (7/12, 58%) than in lower GI defects (3/12, 25%), this numerical difference did not reach significance (p=0.214). No spurting Grade-4 bleeding occurred. The average mucosectomy bleeding severity score in wounds ultimately treated with PuraStat was numerically 11% lower than those treated with Hemospray (1.33 ± 0.65 versus 1.50 ± 0.52), but this was not meaningful (p=0.496).

#### Hemostat delivery

Up to 3 sequential test article applications were allowed within the 10-min observation period to achieve hemostasis (**Table 2**). The mean number of hemostatic treatments (stomach and colon sites combined) was 43% fewer with PuraStat (1.2 ± 0.4 doses/lesion) than Hemospray (1.7 ± 0.8 doses/lesion); this difference approached but did not attain statistical significance (p=0.059). Two Hemospray-treated sites (both stomach; one Grade-1, one Grade-2) were considered treatment failures for rebleeding occurrences after multiple hemostat application during the 10-min evaluation window; however, both sites had stopped bleeding upon relook ≈1h later. Total PuraStat volume delivered was 0.5−1.0 mL per mucosectomy lesion. Hemospray delivery systems were weighed before and after administration to estimate delivered powder mass; however, interpretation is confounded by variable application times and weight loss of the compressed CO_2_ propellant (assumed to begin at 12g per device Instructions for Use). After 1, 2, and 3 applications, Hemospray systems lost 1.6 ± 0.6g (n=6), 10.0 ± 7.8g (n=4), and 14.8 ± 5.8g (n=2), respectively. Weight loss when only a single application was administered ranged from 0.9−2.6g.

#### Time-to-Hemostasis, TTH

In upper-GI mucosectomies average total elapsed TTH ranged from 71−127s after PuraStat treatment and 8−146s after Hemospray treatment (**Table 4**). Upper-GI rebleeding incidence within 10-min after initial wound creation and initial hemostat treatment ranged from 0−1 time with PuraStat and 0−3 times with Hemospray. Two of 12 mucosectomy sites treated with Hemospray were considered failures due to rebleeding that during the final 4 min of the 10-min observation period; these comprised one mild-bleeding defect that received two Hemospray applications and one moderate-bleeding lesion that received 3 Hemospray applications.

**Table 4:** Mucosectomy Bleeding Severity and Time-to-Hemostasis (TTH).

	Bleeding Severity Score	PuraStat		Hemospray	
		Mean TTH, s	# Defects	Mean TTH, s	# Defects
Upper GI	1	88.0	n=4	20.0	n=1*
	2	100.0	n=1	71.0	n=3*
	3	81.0	n=1	−	−
Lower GI	1	52.2	n=5	16.3	n=4
	2	102.0	n=1	161.0	n=2
	3	−	−	−	−
	1	68.1 ± 27.6	n=9	17.0 ± 7.1	n=5
Upper + Lower GI^⁑^	2	101.0 ± 1.4	n=2	111.0 ± 109.8	n=5
	3	81.0	n=1	−	−
All GI Sites/All Bleeding Scores	all	74.7 ± 64.0	n=12	64.0 ± 88.5	n=10*

^*^One Grade-1 (mild bleeding) and one Grade-2 (moderate bleeding) wound in stomachs treated with Hemospray showed delayed-onset rebleeding during the final 4-min window of the 10-min observation period after wound creation/repeated test article application and were considered treatment failures; these samples were excluded from TTH analysis. Bleeding had stopped at both sites upon relook ≈1h later.

^⁑^Average TTH ± SD are shown when n>1; however, values where n=2 are shown for consistency only and are not considered mathematically or biologically meaningful.

In lower-GI mucosectomy lesions, TTH ranged from 30−102s with PuraStat treatment and 8−286s with Hemospray. Note that the longest Hemospray-related TTH of 286s was because the endoscope lens needed cleaning of vision-obstructing hemostatic powder before a 2^nd^ application could be performed; the next longest-duration TTH in this group was 56s. Hemostasis was achieved after 1-2 treatments in both test article groups.

When all mucosectomy sites were combined within each treatment (anatomical locales and bleeding scores) and the two Hemospray-associated failures were excluded, average TTH was similar with PuraStat (74.7 ± 64.0s; n=12) and Hemospray (64.0 ± 88.5s; n=10; p=0.695).

### ESD outcomes

All ESD lesions were able to be created *en bloc*, and measured ≈20 mm in greatest linear dimension, with the exception of a single upper-GI lesion that measured 35 mm and displayed Grade 3 marked bleeding that stopped 39s after application of PuraStat (**Table 5**). Blood flow rates immediately after wound creation in the remaining 5/6 ESD lesions (83%) were categorized as either Mild or Moderate oozing (scoring 1 or 2 points, respectively). Bleeding was stopped in all ESD lesions within 1 min of PuraStat application, with administered volumes ranging from 1.0−5.0 mL.

**Table 5:** ESD Bleeding Severity and TTH.

	Bleeding Severity Score	PuraStat-Treated ESD Lesions			
		Mean TTH, s	# Defects	Defect Size	PuraStat Volume
Upper GI	1	41.0	n=1	20 mm	2.0 mL
	2	95.0	n=1	20 mm	1.0 mL
	3	39.0	n=1	35 mm	4.5 mL
Lower GI	1	75.0	n=1	20 mm	2.0 mL
	2	44.0, 44.0	n=2	20 mm, 20 mm	2.0 mL, 1.0 mL
	3	−	−	−	−
All Sites	−	56.3 ± 23.2s	n=6	22.5 ± 6.1 mm	2.1 ± 1.3 mL

ESD, Endoscopic submucosal dissection; TTH, total elapsed Time-to-Hemostasis.

### Endoscopic relooks

**Figure 2:**
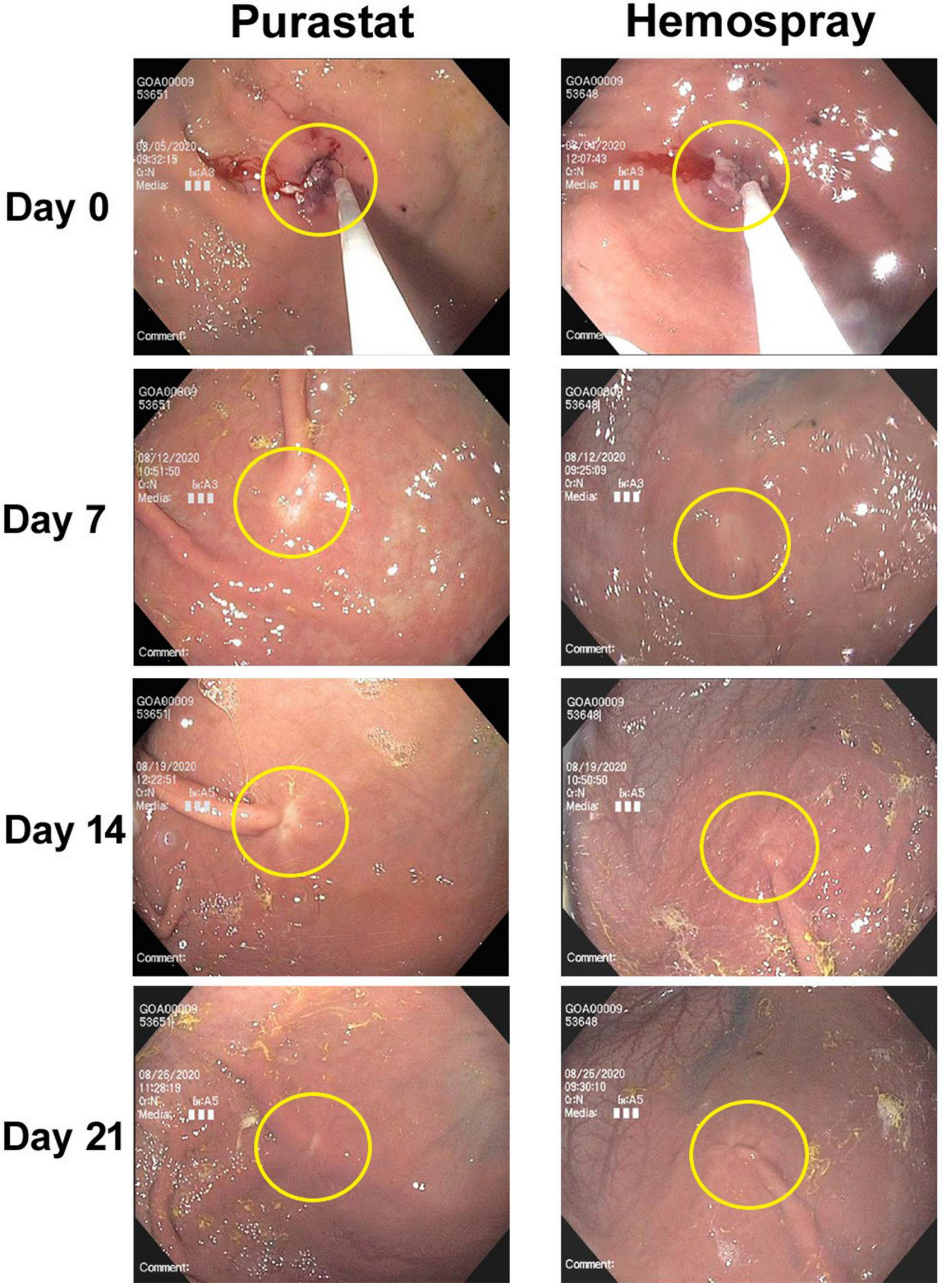
Endoscopic images of mucosectomy defect healing after PuraStat or Hemospray treatment. Shown are 10-mm mucosectomy defects created in the gastric bodies of swine on Day 0, which were treated with either PuraStat self-assembling peptide hydrogel (left) or Hemospray mineral-based aerosol (right) immediately after lesion creation. Good healing is observed by the Day 7 relook, and defect sites are nearly indistinguishable from surrounding unaffected tissues by Day 21.

Endoscopic relook observations were performed on Days 7 ± 1, 14 ± 1, and 21 ± 1 by a single endoscopist (JB). No evidence of delayed bleeding or other significant gross pathological changes to the lesion areas were noted in any animal during relooks. Healing was excellent in both PuraStat- and Hemospray-treated mucosectomy defects, with clear repair obvious by Day 7 and defect areas mostly indistinguishable from unaffected surrounding tissues by Days 21 (**Figure 2**).

### Histology

**Figure 3:**
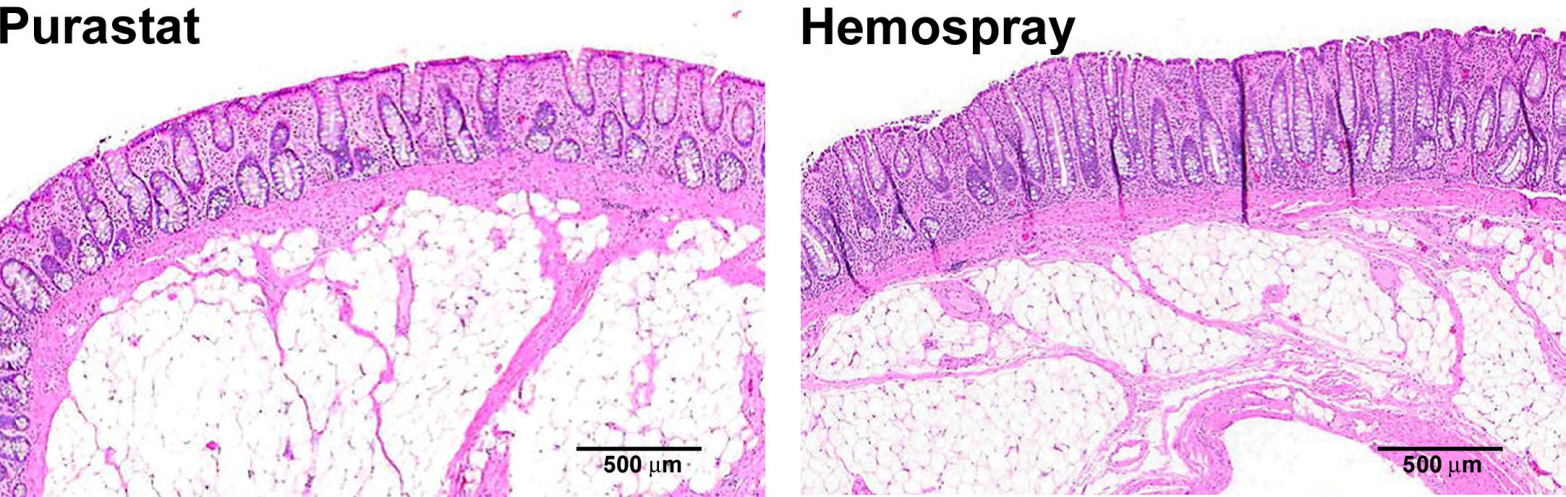
Histology of mucosectomy lesions created in colons and treated with different topical hemostatic agents. Proximal colons of swine underwent 10-cm diameter mucosectomy defect creation followed by immediate topical application of either PuraStat self-assembling RADA16 peptide-based hydrogel (left) or Hemospray mineral-based aerosol (right). Tissues containing lesions were examined at the postoperative 28-day study terminus. Both treatments provided reliable hemostasis of mucosectomy lesions. Healing was similarly good after both hemostat treatments, showing only variable low-level accumulation of inflammatory cells and complete re-epithelialization of the defect area after 1 month of recovery.

On Day 28 ± 1, animals were euthanized and the treated stomach and colon tissues were evaluated. Histologically, neither PuraStat nor Hemospray resulted in any AEs, and both were similarly associated with good healing responses in both stomachs and colons (**Figure 3**). Well-ordered glandular crypts of the mucosa displayed goblet cells containing obvious mucin vacuoles. The lamina propria, muscularis mucosa, and submucosa looked histologically normal after defect recovery treated with either hemostat.

Inflammation scores in surgical defect regions were low and similar in PuraStat- and Hemospray-treated tissues 28 days after lesion creation, in both upper and lower GI lesions (**Table 6**). Untreated lesions displayed numerically higher inflammation scores and rates than defects treated with either hemostatic agent, but only a single test animal was used in this group. Inflammation at postoperative 28 days was heterogeneous, and characterized by the presence of neutrophils (though not seen in PuraStat-treated colon defects), macrophages, lymphocytes, eosinophils, and giant cells.

**Table 6:** Inflammation Scores.

Lesion Site and Treatment	Upper GI (Stomach)		Lower GI (Colon)	
	Score*	Incidence	Score	Incidence
**PuraStat, Mucosectomy (n=12)**	1.17 ± 1.19	67%	1.33 ± 1.12	50%
**Hemospray, Mucosectomy (n=12)**	1.33 ± 0.82	83%	1.33 ± 0.82	83%
**PuraStat, ESD (n=6)**	1.67 ± 1.15	100%	2.33 ± 0.58	100%
**Untreated, ESD (n=4)^¶^**	2.00 ± 0.00	100%	2.00 ± 0.00	100%

*Inflammation Scores ranged from 0 (no inflammatory cells) to 4 (extensive packed sheets of cells)

Untreated lesions were endoscopic submucosal dissections only, not mucosectomies.

### Device performance

PuraStat and Hemospray delivery systems were user-evaluated for acute performance on a Pass/Fail basis. Performance characteristics including satisfactory delivery through the endoscope working channel and trouble-free device performance were assessed as “Passes” for all uses of both the PuraStat (18/18 assessments) and Hemospray (12/12 assessments) systems.

User-friendliness on a 0−4 scale (0=no difficulty; 4=unable to complete procedure) was gauged to be generally excellent with both systems, with one notable exception (**Table 7**). Device Insertion and Device Removal scores were similarly excellent (mean scores <0.5) for both PuraStat and Hemospray systems, with no differences between upper and lower GI tracts. However, the Ability to Treat Intended Site score when accessing and treating upper GI lesions was significantly worse with the Hemospray system (0.83 ± 0.75 points) than with PuraStat (0.00 ± 0.00 points; p=0.008). This disparity was not observed in the lower GI. The PuraStat system scored 0s for all applications. The Hemospray system scored largely 0s, with a few exceptions. Minimal difficulty (Score=1) was experienced in “Device Insertion Ease” into the stomach in a single instance. Minimal difficulty was also logged for “Ability to Treat Site” in 4/12 instances (3 in the stomach), and moderate difficulty (Score=2) was registered in 1/12 instances (stomach). Endoscopist notes indicated that in two instances, repeated Hemospray application was associated with compromised visualization through the endoscope due to adhering opaque hemostat powder, which necessitated mid-procedure scope withdrawal and cleaning before being proceeding.

**Table 7:** Device Performance Evaluation.

Test Device	Hemospray Powder						PuraStat Gel						Powder vs Gel	
Location	Stomach (n=6)			Colon (n=6)			Stomach (n=9)			Colon (n=9)			Stomach	Colon
Usability Parameter	Mean	±	SD	Mean	±	SD	Mean	±	SD	Mean	±	SD	*p*-value	*p*-value
Device insertion	0.17	±	0.41	0.00	±	0.00	0.00	±	0.00	0.00	±	0.00	0.276	0.999
Ability to treat intended site	0.83	±	0.75	0.17	±	0.41	0.00	±	0.00	0.00	±	0.00	**0.008**	0.276
Device removal	0.00	±	0.00	0.00	±	0.00	0.00	±	0.00	0.00	±	0.00	0.999	0.999

Device Usability Scoring:

**0** = Endoscopist User has no difficulty completing procedural step.

**1** = Minimal difficulty resulting in longer procedure or additional instruction required.

**2** = Moderate difficulty resulting in either a much longer procedure or significantly more instruction required.

**3** = Inability to complete the procedural step or damage to the application system.

## Discussion

Achieving prompt and reliable hemostasis in the GI tract is an essential capability when performing endoscopic diagnostic and interventional procedures. Intra-operative bleeding and delayed bleeding/rebleeding are common challenges encountered during endoscopic resectioning of GI lesions or endoscopic interventions for acute GI hemorrhage (25). Current techniques for staunching bleeding in the GI tract include thermocoagulation (*e.g.*, multipolar/bipolar probes, hemostatic forceps, heater probes, argon plasma coagulation, radiofrequency ablation, and cryotherapy), mechanical arrest (*e.g.*, hemoclips, suturing devices, banding devices, stents), or pharmacologic injection treatments (*e.g.*, epinephrine) (2). However, these approaches are associated with potential risk of damaging surrounding tissue, including thermal injury and transmural perforation in extreme cases, and all require unique operator skillsets to accurately and quickly place the hemostatic device directly at the bleeding source. Additionally, epinephrine injection is not recommended for monotherapy because of unreliable hemostasis outcomes, and is associated with rare but serious mural perforation and local tissue necrosis occurrences (26). Topical hemostatic devices, administered *via* catheter through the endoscope working channels, have emerged as minimally-invasive, low-risk means to rapidly achieve durable hemostasis inside the GI tract (2–5).

PuraStat and Hemospray are two commercially-available topical hemostats that, in the current study, were similarly effective at stopping bleeding from experimental GI mucosal lesions. Both hemostatic devices are approved for GI applications (6, 7), and the utility of topical hemostatic agents is recognized in European and U.S. treatment guidelines for treating both upper and lower GI bleeds (27–30). Pigs provide a convenient surrogate for humans in GI endoscopy studies because of close similarities in size and anatomy between the two species; traits that limit the utility of rodent models (16). Topical PuraStat was previously shown to enhance 6-day postoperative neomucosal coverage by 39% and possibly limit the incidence of submucosal damage versus no treatment in a porcine endoscopic GI mucosectomy model (17). Application of the PuraStat peptide to 5-cm circumferential ESD lesions created in pig esophagi facilitated mucosal epithelial regeneration and reduced the incidence of esophageal stricture (18, 19). More recently, monotherapeutic application of a self-assembling peptide with structure similar to PuraStat was evaluated on endoscopically created GI defects in heparinized pigs (20). Hemostasis was achieved within 2 min in 73% of stomach lesions and 80% of duodenal lesions, with no re-bleeding events occurring during a 5-min observation window.

Hemospray has also been extensively tested as a topical hemostat in porcine studies of GI wounding, with variable results. Hemospray provided complete hemostasis (mean 13.8 min) at severe spurting gastric wounds created endoscopically in heparinized pigs by severing gastroepiploic vessels that had been exposed in the stomach lumen through a gastrotomy. Durable hemostasis with no re-bleeding observed after 1 and 24h was achieved in 80% (4/5) treated animals (21). None of the control animals survived >6 h. Necropsy at 1 week demonstrated a healed gastronomy in all Hemospray-treated animals. However, a second study by the same group reported similarly good initial hemostasis outcomes, but 22% of pigs (2/6) exhibited re-bleeds at postoperative Days 8 and 10 (22). In both stomach and rectal mucosal resections performed in 10 pigs, all lesions had decreased in size at postoperative Day 7, but no difference existed between healing in Hemospray-treated and untreated wounds (23). In the current porcine study, both PuraStat and Hemospray afforded good hemostasis outcomes and were associated with appropriate healing in mucosectomy defects. However, two failures to achieve and maintain satisfactory hemostasis during the 10-min observation window after wound creation occurred in the Hemospray group while none occurred in PuraStat-treated lesions. Not counting the Hemospray failures, TTH was similar between groups, averaging 75s in PuraStat-treated mucosectomy sites and 64s with Hemospray. There was a tendency for the endoscopist to repeat fewer device applications with PuraStat than Hemospray. PuraStat was also effective at stopping bleeding from ESD sites in upper and lower GI tracts.

Clinical experiences have shown both PuraStat and Hemospray to be effective topical hemostats for endoscopic control of GI bleeds (3−5). The PuraStat peptide formulation effectively stopped oozing bleeding during endoscopic mucosal resection (EMR) and ESD for stomach tumors, with a mean TTH of 105s and no rebleeding or treatment-related AEs (31). In 56 patients undergoing EMR and ESD for diverse upper and lower GI lesions, PuraStat-mediated hemostasis had a 6% delayed bleed rate even though 45% of lesions were categorized as high-risk for rebleeding (32). A mean 3.5 mL of PuraStat was applied per lesion, compared to 2.1 mL used for ESD lesions in the current study. No treatment-related AEs occurred. In 51 lesions of 45 patients undergoing gastric ESD, application of a 1% solution of the PuraStat peptide provided good hemostasis with a 2% rebleeding rate through 6 weeks follow-up (33). No treatment-related AEs were observed. A study of 100 patients who underwent complex endoscopic resection (21 EMR and 79 ESD) in various upper and lower GI regions, with a large resection area (mean 14 cm^2^), PuraStat was an effective hemostat when used as monotherapy in 73% (45/62) of total attempts, including in 50% (3/6) of spurting bleeds (34). Mean TTH in that study was 70s, and the delayed bleeding rate was 3%. An average 1.8 mL of PuraStat per lesion was used to control intraprocedural bleeding, and 2.6 mL to cover the resection base. No treatment-related AEs were observed. In 101 subjects undergoing esophageal or colorectal ESD who were randomized to thermocoagulation with or without adjunctive PuraStat treatment for hemostasis, peptide hydrogel therapy was associated with a 50% reduction in diathermy use, and the rebleeding rate was ≈4% in both groups (11). A retrospective observational study evaluated endoscopically applied PuraStat as rescue therapy in 77 patients with acute GI bleeds (41 upper GI, 36 lower GI) (35). Bleed types were defined 83% oozing and 17% spurting; 65% were iatrogenic (56% associated with EMR), and non-iatrogenic bleeds were primarily peptic ulcer (25%) and cancer (7%). Immediate hemostasis was achieved in 90% of all cases using PuraStat, including approximately half (6/13) of the spurting bleeds. The rebleeding rate through 7 days was 10%, and 6/8 patients with rebleeding achieved hemostasis after PuraStat reapplication, with the remainder requiring surgery. Another recent prospective study of 111 subjects with acute non-variceal GI bleeding (upper=30%, lower=70%) reported 94% hemostatic success using PuraStat as the primary approach, and 75% when used in a secondary fashion after failure of other techniques (4). Rebleeding rates through 3 and 7 days were 9% and 13% respectively, after primary use. The overall 30-day rebleeding rate through 30 days was 16%. No AEs were reported. Thus, although PuraStat is primarily indicated for stopping oozing bleeding, it can be useful as an adjunctive therapy for some acute spurting GI lesions. In the current study, PuraStat was an effective hemostat for upper and lower GI wounds displaying oozing bleeding of mild-to-moderate severity.

Hemospray has also been extensively evaluated as a topical hemostat in the clinical GI endoscopy setting, and several reviews of study outcomes have been published (3, 9, 10, 36–38). A 2015 narrative review of 17 publications from 2011−2014 involving 234 subjects who endoscopic GI treatment with Hemospray for diverse bleeding causes suggested an 88.5% initial hemostasis (clinical success) rate, with a 16.2% rebleed occurrence through 72 h (9). The reviewed publications included 13 case series and 6 single-case reports. A 2019 systematic review of Hemospray use for upper GI bleeds evaluated 50 studies published up to October 2018 involving 1445 subjects; 28 reports (56%) were conference abstracts and 22 (44%) were full-text papers; 42 reports (84%) were case series, 4 (8%) were clinical trials, and 4 (8%) were cohort studies (36). They identified a 90.7% primary hemostasis rate and a 26.1% aggregate rebleeding rate (follow-up range 12h to 3 months). Altogether, only 5 device-related AEs were reported, comprising 1 perforation and 4 cases of endoscope adhesion to the gastric cardia when Hemospray was applied in retroflexed view; however, there were 11 articles in which AEs were not addressed. Similar upper GI findings were reported in a 2020 meta-analysis of 20 studies involving 1280 subjects who received Hemospray for non-variceal upper GI bleeding (10). In this assessment, clinical success rate was 91%, and aggregate rebleeding rate (all causes and times after initial hemostasis achievement) was 27%, including a 20% early (≤72h from successful initial treatment) rebleed rate and a 9% delayed (>72h) rebleed rate (10). Nine patients (0.7%) experienced 12 total AEs following Hemospray use, of which 9 were device-related (8 gastrointestinal perforations and 1 report of abdominal pain). In the lower GI tract, Hemospray outcomes reported in a 2021 systematic review of 8 studies (175 subjects) largely involving oozing post-polypectomy bleeds, include a 96.2% initial hemostasis success rate, and a 9.8% 7-day rebleeding rate and 12.3% 30-day rebleeding rate (37). Adverse events were limited to two cases of mild postoperative abdominal pain. An updated 2021 review of Hemospray GI outcomes of 27 studies (1916 subjects) published through January 2019 reported a similar clinical success rate of 94.5%, respective 7- and 30-day rebleeding rates of 9.9% and 17.6%, and total AE incidence of 0.7% (38). When comparing conventional therapy to Hemospray-augmented hemostasis, although initial hemostasis outcomes were good with Hemospray, its inclusion was not associated with improvement in 8-day rebleeding rate or 30-day mortality. This study included both upper and lower GI studies, with an expected preponderance of upper GI outcomes. Thus, while Hemospray is effective in achieving immediate hemostasis in the GI tract it is associated with a significant rate of rebleeding, particularly in the upper GI tract in which large lesion sizes and spurting bleeding are more common than in the lower GI tract, potentially compounded by the acidic gastric environment. Rare but potentially serious cases of transmural perforation have occurred with the pressurized gas Hemospray delivery system that have not been reported with the PuraStat liquid applicator.

The biocompatibility of both PuraStat and Hemospray was confirmed by good mucosal healing through 4 weeks after treatment with either system, with good re-epithelialization and mucosal regeneration and only minimal evidence of lasting inflammation at the study endpoint. A prior study similar to ours reported no effect of Hemospray on healing rate of experimental mucosectomy lesions created in stomachs and rectums of pigs, though outcomes were only tracked through 7 days after treatment (23). In the current porcine study, histological inflammation scores were similarly good with PuraStat and Hemospray at the 4-week terminus, and did not significantly differ between upper and lower GI lesions. No treatment-related AEs occurred with either hemostat device.

Technical success is an essential parameter when attempting topical GI hemostasis. Both PuraStat and Hemospray share important utility in being able to cover large lesions that might be encountered with peptic ulcers or at neoplastic excision sites. Additionally both systems benefit from touch-free hemostat application without the catheter directly contacting the GI mucosal lining; this reduces the risk of accidental mural perforation by the delivery catheter tip. However, high-pressure powder and propellant flow from the compressed CO_2_-driven Hemospray device may introduce risk of rare but potentially serious mucosal perforation events if the catheter tip is closely juxtaposed to the GI tract lining (9). We are uncertain if this possibility is related to the manufacturer’s modification of the Hemospray system that reduced CO_2_ pressurization from the original 55 psi value down to 37 psi (39).

Clear visualization of the surgical field is essential to accurately performing endoscopic procedures. One advantage of the PuraStat formulation is the supple transparent barrier formed upon hydrogel self-assembly allows clear visualization of the underlying defect site, and additional interventional measures such as hemoclip application can be performed through the PuraStat layer without encountering physical resistance. By contrast, the opaque nature of the mineral-based Hemospray barrier may obscure anatomical landmarks on the GI wall and hinder undertaking additional hemostatic measures if needed (9). Any unwanted treatment delay might have serious health outcome implications in the case of severe acute GI bleeds. We encountered two incidents in which the endoscope lens became coated with Hemospray powder mid-procedure, necessitating its withdrawal and cleaning before continuing treatment; surgical site visualization remained excellent throughout all PuraStat applications.

Operator satisfaction and assessment of device performance were rated favorably for both systems, and no critical device failures occurred. However, the PuraStat system had a significantly better mean Ability to Treat Intended Site score for stomach sites than did the Hemospray system, due to the reporting of minimal-to-moderate difficulty in accessing stomachs and treating lesions with the Hemospray system in 25% of cases. In a review of 50 Hemospray upper GI studies, equipment failures were reported in 8 studies (36). Of 427 applications, 21 events (4.9% incidence) were identified comprising catheter obstruction by powder (n=19), CO_2_ cartridge malfunction (n=1), and endoscope working channel occlusion by aspirated powder (n=1); in most cases, the problem was resolved by replacing the application catheter [systems are distributed with two catheters (6)]. Utility of the PuraStat delivery system has also received good evaluations in prior studies (32–34).

PuraStat and Hemospray safely provided similarly effective hemostasis in this porcine model of upper and lower GI endoscopic wounding, with no adverse reactions. Study strengths include the use of an established large-animal GI endoscopy model and, to our knowledge, a prior lack of direct comparisons between PuraStat and Hemospray outcomes in GI hemostasis. A primary study limitation was the low number of untreated control animals that precluded detailed statistical comparison of bleeding outcomes and tissue healing to the two active comparators. Additionally, our study design allowed comparison of hemostasis and mucosal healing/lesion histology parameters in mucosectomy lesions after PuraStat versus Hemospray treatment, but not in ESD lesions. While findings from an animal study cannot be directly translated to the human condition, our results are all in accord with those of prior clinical trials. We conclude that PuraStat and Hemospray, while being clearly distinct mechanochemical formulations with unique trans-endoscopic delivery platforms, are both safe means to provide effective hemostasis and support wound healing in diverse GI defects.

## Data availability statement

The raw data supporting the conclusions of this article will be made available by the authors, without undue reservation.

## Ethics statement

The animal study was reviewed and approved by the CBSET Inc., Contract Research Organization’s (Lexington, MA) Institutional Animal Care and Use Committee (IACUC), and conformed to the “Guide for the Care and Use of Laboratory Animals, 8th edition” and the ARRIVE 2.0 Guidelines. CBSET is an AAALAC International accredited facility and is registered with the U.S. Department of Agriculture.

## Author contributions

All authors listed have made a substantial, direct, and intellectual contribution to the work, and approved it for publication.

## Funding

This study was funded by 3-D Matrix Inc., Newton, MA, the manufacturer of the synthetic peptide agent used in this study. The funder was not involved in the study design, collection, analysis or interpretation of the data, the writing of this article, or the decision to submit it for publication.

## Acknowledgments

The authors thank Matthew Silverman, PhD (Biomedical Writing Solutions, Wakulla Springs, FL) for scientific, statistical, and writing assistance. Doctor Silverman’s fees were paid by 3-D Matrix Inc., Newton, MA.

## Conflict of interest

Authors EG and EA were employed by 3-D Matrix Inc.,Newton, MA, United States. Author KO’N was employed by 3-DMatrix Ltd., London, United Kingdom. Authors JB and RM were employed by CBSET Inc. Author LS was employed by First Edge Consulting LLC.

## Publisher’s note

All claims expressed in this article are solely those of the authors and do not necessarily represent those of their affiliated organizations, or those of the publisher, the editors and the reviewers. Any product that may be evaluated in this article, or claim that may be made by its manufacturer, is not guaranteed or endorsed by the publisher.
